# Diverse traits of aquatic plants cannot individually explain their consumption by the generalist gastropod *Biomphalairia glabrata*

**DOI:** 10.7717/peerj.12031

**Published:** 2021-09-20

**Authors:** Renato Crespo Pereira, Nathália Nocchi, Tatiana U.P. Konno, Angelica R. Soares

**Affiliations:** 1 Universidade Federal Fluminense, Departamento de Biologia Marinha, Rio de Janeiro, Brazil; 2 Instituto de Pesquisas Jardim Botânico do Rio de Janeiro, Rio de Janeiro, Brazil; 3 Instituto Universitario de Bio-Orgánica Antonio González, Departamento de Química Orgánica, Universidad de La Laguna (ULL), La Laguna, Tenerife, Spain; 4 Grupo de Produtos Naturais de Organismos Aquáticos, Instituto de Biodiversidade e Sustentabilidade (NUPEM), Universidade Federal do Rio de Janeiro, Macaé, Rio de Janeiro, Brazil; 5 Instituto de Biodiversidade e Sustentabilidade (NUPEM)/Grupo de Produtos Naturais de Organismos Aquáticos, Universidade Federal do Rio de Janeiro (UFRJ), Macaé, Rio de Janeiro, Brazil

**Keywords:** Herbivory, Aquatic biology, Macrophytes, Chemical defense, Physical defense, Structural defense, Secondary metabolites, Plant life-form, Plant defense, Susceptibility to herbivory

## Abstract

Several experimental studies on aquatic plants have reported the prevalence of chemical defense mechanism against herbivory, as opposed to structural, life-forms or other traits. Here, our laboratory feeding experiments and integrative analysis explored the relationship among palatability (fresh or reconstituted plants used as artificial diet) and various chemical/nutritional traits (*i.e.,* contents of dry mass, ash, nitrogen, protein, and phenols) of diverse aquatic plants and their susceptibility to consumption by the generalist gastropod *Biomphalaria glabrata*. *Biomphalaria glabrata* consumed all of the assayed aquatic plants in a hierarchical yet generalized way, with the consumption of fresh plants, their reconstituted forms and defensive properties of lipophilic extracts not being significantly correlated with plant physical or chemical traits to determine the feeding preference of the gastropod. Our results do not reveal a prevalence for a specific plant attribute contributing to herbivory. Instead, they indicate that the susceptibility of aquatic plants to generalist consumers is probably related to a combination of their chemical and physical properties, resulting in moderate grazing rates by generalist consumers.

## Introduction

Herbivory has long been recognized as a selective and structuring component in terrestrial (Hairston, Smith & Slobodkin, 1960) and marine environments (Lubchenco, 1978). Although recognized later, herbivory on aquatic plants is also considered selective and intense (Newman, 1991; Lodge et al., 1998) and it is capable of removing about 44–48% of plant biomass, exerting cascade effects on community structure in aquatic environments (Bakker et al., 2016). Terrestrial and aquatic environments share several structural features, but the ecology and evolution of chemical defense and other components of plant-herbivore interactions remain poorly understood in freshwater environments.

Aquatic plants are known to produce a wide variety of secondary metabolites, such as phenols, terpenes, alkaloids, polyketides, among others (Ramesh, Rajan & Santhanan, 2014). Give the selective pressure of herbivory (Lodge, 1991) and the considerable diversity of defensive metabolites (Ramesh, Rajan & Santhanan, 2014), have compounds likely play important ecological roles in freshwater environments. Indeed, diverse studies, from simple defensive chemical content analysis (*e.g.*, Ostrofsky & Zettler, 1986) to more complex experimental studies (*e.g.*, Bolser & Hay, 1998) support that likelihood.

In one of the first such experimental studies, extracts of aquatic plant species were shown to significantly reduce herbivory (Bolser et al., 1998). Extracts of two species of aquatic plants, *Nuphar luteum* var. *macrophyllum* and *Alternanthera philoxeroides*, were found to inhibit the consumption by the omnivorous crayfish *Procambarus clarkii*, though other aspects, such as structure and nutritional content also contributed to the consumer’s food selectivity (Cronin et al., 2002). Defensive chemicals in the aquatic plant species *Micranthemum umbrosum* were shown to inhibit consumption by the crustaceans *Procambarus spiculifer* and *P. acutus*, as well as the fish *Ctenopharyngodon idella* and these chemically-mediated relationships are structuring for the freshwater plant community (Parker et al., 2006).

In the context of allelopathic defense, chemicals in the exudates of *Eichhornia crassipes* and *Salvinia biloba* repel the cladocera *Ceriodaphnia dubia* and decrease its life expectancy, increasing its age at first reproduction and lowering the number of eggs generated per female (Gutierrez & Paggi, 2014). Chemical exudates from aquatic plants also repel some planktonic organisms (Pennak, 1973; Dorgelo & Heykoop, 1985).

Chemical defense by aquatic plants can also be dynamic or induced upon perceiving a cue that indicates an increased risk of the plant being grazed. The specialist beetle *Galerucella nymphaeae* induces chemical defense in the water lily *N. luteum* var. *macrophyllum* that inhibits consumption by the generalist crayfish *P. clarkii* (Bolser & Hay, 1998). Chemical defense in *Myriophyllum spicatum* induced by the activity of the insect *Acentria ephemerella* promotes protection of the apical regions of this plant, preventing future attacks by this same species (Fornoff & Gross, 2014). In *Cabomba caroliniana*, chemical defense is induced by *P. clarkii* and by the snail *Pomacea canaliculate*, which reduces this plant’s palatability of this plant for both consumers and also suppresses the growth of co-occurring microbes (Morrison & Hay, 2011). Rapid production of nitrogen in apical regions of *M. spicatum* upon consumption by the herbivorous insect *Acentria ephemerella* supports the existence of a nitrogen-based chemical defense system in these young plant parts (Rothhaupt, Fornoff & Yohannes, 2015).

A review highlighted that the chemical defenses in aquatic plants are extensive but remain largely unexplored (Gross & Baker, 2012). There is little quantitative information on what determines the susceptibility of aquatic plants to herbivory or the basis of choice by herbivores (Lodge, 1991; Newman, 1991). However, several experimental studies support an apparent prevalence of chemical defense over other plant traits in herbivore feeding preferences (*e.g.,* Kubanek et al., 2001; Gutierrez & Paggi, 2014).

Although various structural plant defenses, such as spines, thorns, or pubescence/hairiness are rare or even non-existent in aquatic plants (Vermeij, 2016), few structural aspects of such plants have been implicated in the feeding preferences of freshwater herbivores (Chambers, Hanson & Prepas, 1991; Cronin, 1998). Instead, plant nutritional value (Fraser, Chavez & Paloheimo, 1984; Doucet & Fryxell, 1993; Kreider & Watts, 1998) and presence of chemical defense are widely considered the main traits determining aquatic plants palatability (Cronin et al., 2002; Sotka et al., 2009). However, there is still a lack of integrated studies exploring all aspects contributing to plant-herbivore interactions in freshwater environments and that can elucidate the ecology and evolution of these trophic relationships.

Here we deployed an integrated experimental approach to investigate the relationship between the susceptibility of diverse aquatic plant life-forms, from emergent and floating to fully submerged species, and several of their physical and chemical traits in terms of their consumption by the generalist gastropod *Biomphalaria glabrata*.

## Materials & Methods

### Field sites and sample collections

Thirteen aquatic plant species belonging of different families and that represented a wide range of life-forms (according Cook, 1996) and taxonomic groups were target (Table 1). All plants were collected in aquatic habitats inside the Restinga de Jurubatiba National Park, Rio de Janeiro State, Brazil (SISBio 30299). Immediately after collection, the plants were conditioned in thermal boxes and transported to the laboratory. The species were identified by one of the authors (Tatiana U.P. Konno) and voucher specimens have been deposited at the Herbarium of the Rio de Janeiro Federal University, Brazil. Samples of vegetative parts of each species were separated into three groups: (1) fresh plants for use in susceptibility to consumption assays (within 48 h of collection); (2) lyophilized and ground for the assays using reconstituted plants (as artificial food) and the nutritional analyses; (3) chemical extractions to investigate the defensive capabilities.

**Table 1:** Information on aquatic plant species used in the assays, including family, habit (life-form), collected site coordinates, and voucher number.

**Family**	**Species**	Habit (life-form^*^)	**Collection site**	**Voucher**
Alismataceae	*Hydrocleys nymphoides* (Willd.) Buchenau	Hydrophyte: emergent free floating	22°17′S 41°41′W	RFA39121
Araceae	*Pistia stratiotes* L.	Hydrophyte: emergent free floating	22°17′S 41°42′W	RFA39122
Cyperaceae	*Eleocharis interstincta* (Vahl) Roem. & Schult.	Hydrophyte: emergent	22°17′S 41°41′W	RFA 39123
Lentibulariaceae	*Utricularia foliosa* L.	Hydrophyte: submerged	22°17′S 41°41′W	RFA39244
Menyanthaceae	*Nymphoides humboldtiana* (Kunth) Kuntze	Hydrophyte: emergent floating	22°12′S 41°29′W	RFA39117
Nymphaeaceae	*Nymphaea pulchella* (Salisb.) DC.	Hydrophyte: emergent floating	22°17′S 41°41′W	RFA39245
Pontederiaceae	*Eichhornia azurea* (Sw.) Kunth	Hydrophyte: emergent floating	22°17′S 41°41′W	RFA39120
Potamogetonaceae	*Potamogeton illinoensis* A. Benn.	Hydrophyte: emergent floating	22°16′S 41°39′W	RFA41421
Pteridaceae	*Ceratopteris thalictroides* (L.) Brongn.	Hydrophyte: emergent	22°17′S 41°41′W	RFA39211
Rubiaceae	*Oldenlandia salzmannii* (DC.) Benth. & Hook. f. ex B.D. Jacks.	Hydrophyte: submerged to Helophyte	22°16′S 41°39′W	RFA39119
Salviniaceae	*Salvinia auriculata* Aubl.	Hydrophyte: emergent free floating	22°16′S 41°39′W	RFA39242
Typhaceae	*Typha domingensis* Pers.	Fixed emergent	22°17′S 41°41′W	RFA39234
Xyridaceae	*Xyris brevifolia* Michx.	Helophyte: submerged	22°10′S 41°31′W	RFA39240

**Notes.**

* Life-forms according to Cook (1996).

Specimens of the generalist herbivorous gastropod *B. glabrata* (Thomas, Nwanko & Sterry, 1985) (shell diameter = 1.7 ± 0.3 cm) were collected from Cabiúnas lagoon (22°16′S, 41°41′W) by actively searching through clumps of aquatic plant using a sieve. Upon collection, the gastropods were conditioned in thermal aerated boxes containing freshwater and transported to the laboratory. There, they were maintained in plastic containers (10 L) with freshwater changed daily, constant aeration, constant temperature (22 °C) and a 12:12 h light:dark photoperiod. They were fed on lettuce for 48 h until 1 h before the beginning of experiments to avoid starvation that could promote unnaturally feeding behavior during our assays (Cronin & Hay, 1996).

### Susceptibility of the fresh whole aquatic plants to *B. glabrata*

The susceptibility of nine species of aquatic plants to consumption by *B. glabrata* was assessed according to a previous study (Cronin et al., 2002). Each experimental unit (replicate) consisted of a transparent plastic container (1 L) supplied with non-recirculating freshwater, in which one sample (approximately equal volumes to provide comparable encounter rates) of each of the nine types of aquatic plant was simultaneously offered to a single individual of *B. glabrata* for 3 days (*n* = 14) to determine the differential susceptibility of them to this gastropod. Only vegetative parts of each of the nine plant species were used. One sample of each plant species was set up under identical conditions, but without *B. glabrata* (as a control) to verify possible autogenic changes in biomass (independent of herbivory). Each plant sample was weighed at the beginning and at the end of the experiment, and the biomass consumed by *B. glabrata* was expressed as a percentage (%) of consumption, calculated according to the equation [H_0_ ×(C_f_/C_0_) −H_f_] ×100; where H_0_ and H_f_ represent the biomass of the plant sample exposed to herbivory before and after the assay, respectively, and C_0_ and C_f_ represent the biomass of corresponding control samples of the species from before and after the assay. For this bioassay, 14 replicates of each plant species were used.

### Consumption of reconstituted aquatic plants by *B. glabrata*

To determine if plant structure influenced the consumption by *B. glabrata*, samples of the 13 collected aquatic plant species were frozen, freeze-dried, ground into a fine powder, and stored at −4 °C for later preparation as artificial foods. Artificial foods (*i.e.,* reconstituted plants) were prepared in agar gel to reproduce the natural dry mass concentration of each species, as described previously (Bolser & Hay, 1998; Cronin et al., 2002). The amount of powdered plant incorporated as artificial foods varied for each species due to the specific relationships between dry mass and wet mass. The artificial food comprising each plant species was prepared by mixing 5.0 mL of distilled water with 0.36 g of agar, heating the mixture in a microwave oven until boiling, and then pouring it into cold water containing desired amounts of freeze-dried powder for each species (*i.e.,* each species was rehydrated with the same amount of cold water to reestablish its wet mass). This mixture was allowed harden onto a plastic screen mesh (∼1 mm^2^ per aperture) and flattened between two layers of wax paper. After cooling and solidification, the screen mesh was cut into smaller pieces of 10  × 10 mm^2^ (total of 100 squares). Accordingly, all artificial foods presented the same morphology and hardness, but the original nutritional value and taste of each species was maintained as nuch as possible. For this bioassay, 30 replicates of each plant species were used.

The 13 artificial foods were simultaneously offered to one specimen of *B. glabrata* in 1.0 L plastic containers supplied with non-recirculating freshwater (*n* = 30) and consumption of them was calculated by scoring the number of squares of artificial food that were consumed. A fixed consumption stopping rule was applied (Lockwood III, 1998), where all assays were termined once >50% of all artificial food had been consumed.

### Defensive capacity of the plant extracts against *B. glabrata*

The defensive capabilities of the extracts from ten aquatic plants against *B. glabrata* were evaluated by assaying *B. glabrata*’s consumption of an artificial food containing the extract (treatment) and a respective control (without extract). A lipophilic extract of each plant species was incorporated at a naturally occuring concentration (extract weight equivalent to the plant dry weight) to artificial food (treatment and control) prepared as described previously (Bolser & Hay, 1998; Cronin et al., 2002). For treatment foods, lipophilic aquatic plant extracts were first dissolved in CH_2_Cl_2_, added to a freeze-dried lettuce:broccoli mixture (1:1) that lacks defensive compounds, and then the solvent was removed by rotary evaporation. This procedure resulted in a uniform coating of the lipophilic extracts on the lettuvce:broccoli plant particles (Hay, Kappel & Fenical, 1994). For the control food, only the solvent was incoprporated into the lettuce:broccoli mixture. Artificial foods (treatment or control) were then prepared by adding 0.36 g of agar to 5.0 mL of distilled water, heating the mixture in a microwave oven until boiling, and then pouring it into 2 mL of cold water containing 0.5 g of freeze-dried powder of the lettuce:broccoli (1:1) mixture with (treatment) or without (control) extract. Treatment and control artificial foods were hardened separately onto a plastic screen mesh (∼1 mm^2^ per aperure) and flattened between two layers of wax paper. After the artificial foods had cooled and solidified, the screen mesh was cut into strips comprising 100 squares (10 mm^2^) of artificial food pieces of 10  × 10 mm square (control and treatment). The feeding assays involved simultaneously offering a treatment (one particular plant extract) and a control strip of artificial food to one individual *B. glabrata* as a two-way choice. The assays were carried out in small plastic aquaria (500 mL) containing 250 mL of the water collected from Cabiúnas Lagoon. The number of replicates for each extract (species) varied between 18 and 26, and gastropod specimens were not used for more than one replicate.

Food consumption was estimated as described above under the section *Consumption of reconstituted freshwater plants by B. glabrata*, and the fixed-consumption stopping rule was again applied. Percentage inhibited consumption was was calculated according to the differences between consumption of control and treatment artificial foods, quantified as % Inhibition = [(C –T)/C] ×100 for each replicate, where C and T correspond to control and treatment squares consumed, respectively.

### Extracts preparation

Extracts were obtained from a total of 10 aquatic plant species. Their vegetative parts were separated and air-dried under shade and in a climatized room at ambient temperature (mean 21 °C) for 48 h. In order to minimize the intra-individual variability among organisms, each plant was gathered and subjected to extraction together. Dried plants (*X*. *brevifolia*, 47.7 g of dry weight, d.w.; *E. interstincta* 27.3 g d.w.; *T. domingensis* 26.3 g d.w.; *P. illinoensis* 19.6 g d.w.; *N. humboldtiana* 95.0 g d.w.; *E. azurea* 54.7 g d.w.; *S. auriculata* 31.3 g d.w.; *P. stratiotes* 42.0 g d.w.; *O. salzmannii* 29.8 g d.w.; *U. foliosa* 193.0 g d.w.) were ground down and extracted with a mixture of dichloromethane:methanol, 1:1 (HPLC grade, Tedia), using 20 mL of solvent for each 1 g of plant dry mass for one day at room temperature. Then the solvent was filtered out and the biological material was re-extracted using the same solvent/plant mass ratio. This procedure was repeated twice, and then the solvents removed from these extractions were combined and dried by rotary evaporation (Buchi R210, V850, B491, Switzerland) to yield a total of 10 different plant extracts that were used in the feeding assays and to quantify phenolic contents. The extract from *Potamogeton illinoensis* was further subjected to chemical profile analysis by Proton Nuclear Magnetic Resonance (^1^H NMR).

### Physical, nutritional and chemical properties of the aquatic plants

Several tissue properties known to influence herbivory were measured for the 13 collected aquatic plant species. Powdered aquatic plants were analyzed for total nitrogen using the Kjeldahl method, as described previously (Conklin-Brittain et al., 1999), and the protein content was estimated by multiplying the value for total nitrogen by a factor of 6.25 (see Barbarino & Lourenço, 2005). Ash content was accurately determined *via* combustion of powdered biomass in a muffle oven at 550 °C (Jones, 2001). Lipids were extracted with diethyl ether using a Soxhlet system (see details in Ramluckana, Moodley & Bux, 2014). Results for ash and lipids contents are expressed as a percentage (%) by dry mass, total nitrogen as g% nitrogen (N) and protein content as g% protein (P). Phenol content was quantified from crude extracts of the plants using a modified Folin-Ciocalteu colorimetric method (Zhang et al., 2006) and the results are expressed as milligrams of GAE (gallic acid equivalent)/g of the extract. All analyses were performed in triplicate.

### Chemical profile of *Potamogeton illinoensis* by ^1^H NMR

The chemical profile *P. illinoensis*, the most active extract against *B. glabrata*, was analyzed by ^1^H NMR in order to identify its major lipophilic compounds. NMR analysis was performed on the Varian VNMRS apparatus with 11.75 T (500 MHz) magnet. Deuterated chloroform was used to solubilize the sample.

### Data analysis

To evaluate the various relationships between the consumption by *B. glabrata* and the different plant traits, data on consumption and the various physical, nutritional and chemical properties of the plant species s were compared according to species and also plant life-forms.

Non-parametric tests were used to analyze the herbivory data, as data was not show normally distributed. To compare among species, data on percentage consumption (%) whole plants and artificial foods (reconstituted plants) by *B. glabrata* were analyzed using the non-parametric Friedman test due data dependency (all species were offered simultaneously to the herbivore). Therefore, the comparison between the different life-form groups the % of consumption data of the whole and reconstituted aquatic plants were analyzed by Kruskal-Wallis non-parametric analysis of variance due to inequality in the sample size of the groups. For pair-wise comparisons of the non-parametric tests, the post-hoc Durbin-Conover and Dwass-Steel-Critchlow-Fligner test were performed to the Friedman and Kruskal-Wallis analysis, respectively. One Way ANOVA, followed by a post-hoc Tukey test was applied for comparisons of physical, nutritional and chemical properties of the aquatic plants. Pearson correlation coefficients were used to evaluate relationships among percentage consumption of whole plants, artificial reconstituted plants, extract-mediated inhibition of consumption, and various physical, nutritional and chemical traits of the plant species. For all statistical analyses, results were considered significant when *p* < 0.05. All statistical analyses of variance and correlation were performed in Jamovi version 1.6 freeware (R Core Team. 2020; Jamovi The Project, 2021).

A multivariate approach was conducted using the exploratory descriptive Principal Components Analysis (PCA) to summarize all data and allow access and visualization of a possible relationship between plant traits and herbivory inhibition. The consumption data, and physical, nutritional and chemical traits were summarized in a matrix that was submitted to a PCA. The physical, nutritional and chemical traits were used as supplementary quantitative variables. The PCA was performed in R version 4.0.3 with the function “PCAshiny” in the “Factoshiny” package (Vaissie, Monge & Husson, 2021). The scores results were represented by scatter plots of the principal components (PC) for visualization of the discriminant pattern of the samples (species and plant life-form) according to their distribution along the first two PC axes (PC1 =Dim 1 and PC2 =Dim 2). The loading results (consumption data, physical, nutritional and chemical traits) used to discrimination of the samples were represented by scatter plots.

## Results

### Susceptibility of intact plants to *B. glabrata* herbivory

**Figure 1:**
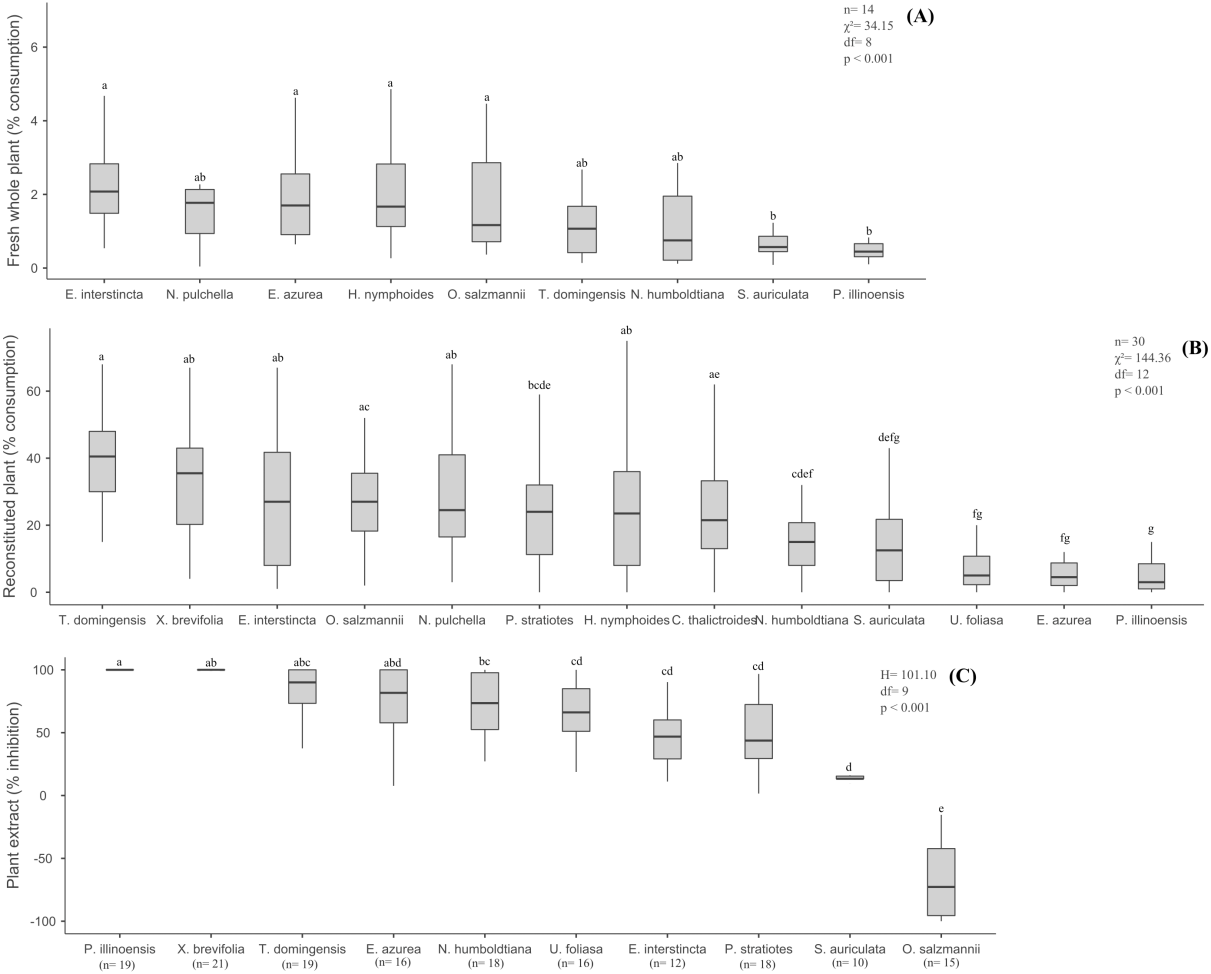
Results of the choice feeding assays with *B.glabrata*: (A) Susceptibility of nine fresh whole plants; (B) consumption of reconstituted thirteen plants; and (C) defensive effect of ten plant extracts. The same letters on bars indicate the treatments are not significantly different. Bars represent mean (± standard deviation). Lower and upper box boundaries 25th and 75th percentiles, respectively, line inside box median and lower and upper error lines 10th and 90th percentiles, respectively.

The percentage of fresh biomass of the nine aquatic species consumed by *B. glabrata* ranged from 2.08% (*E. interstincta*) to 0.45% (*P. illinoensis*) (median values, Fig. 1A), revealing three groups, two of which differed significantly from each other (*χ*^2^ = 34.15, *df* = 8, *p* < 0.001, Tukey test). All plant species were consumed to some degree, but with the following hierarchy in terms of diminishing preference (Fig. 1A): *E. interstincta* > *N. pulchella* > *E. azurea* > *H. nymphoides* (group 1) > *O. salzmannii* > *T. domingensis* > *N. humboldtiana* (intermediate group, not significantly different from groups 1 and 2, *p* > 0.05) > *S. auriculata* > *P. illinoensis* (group 2).

**Figure 2:**
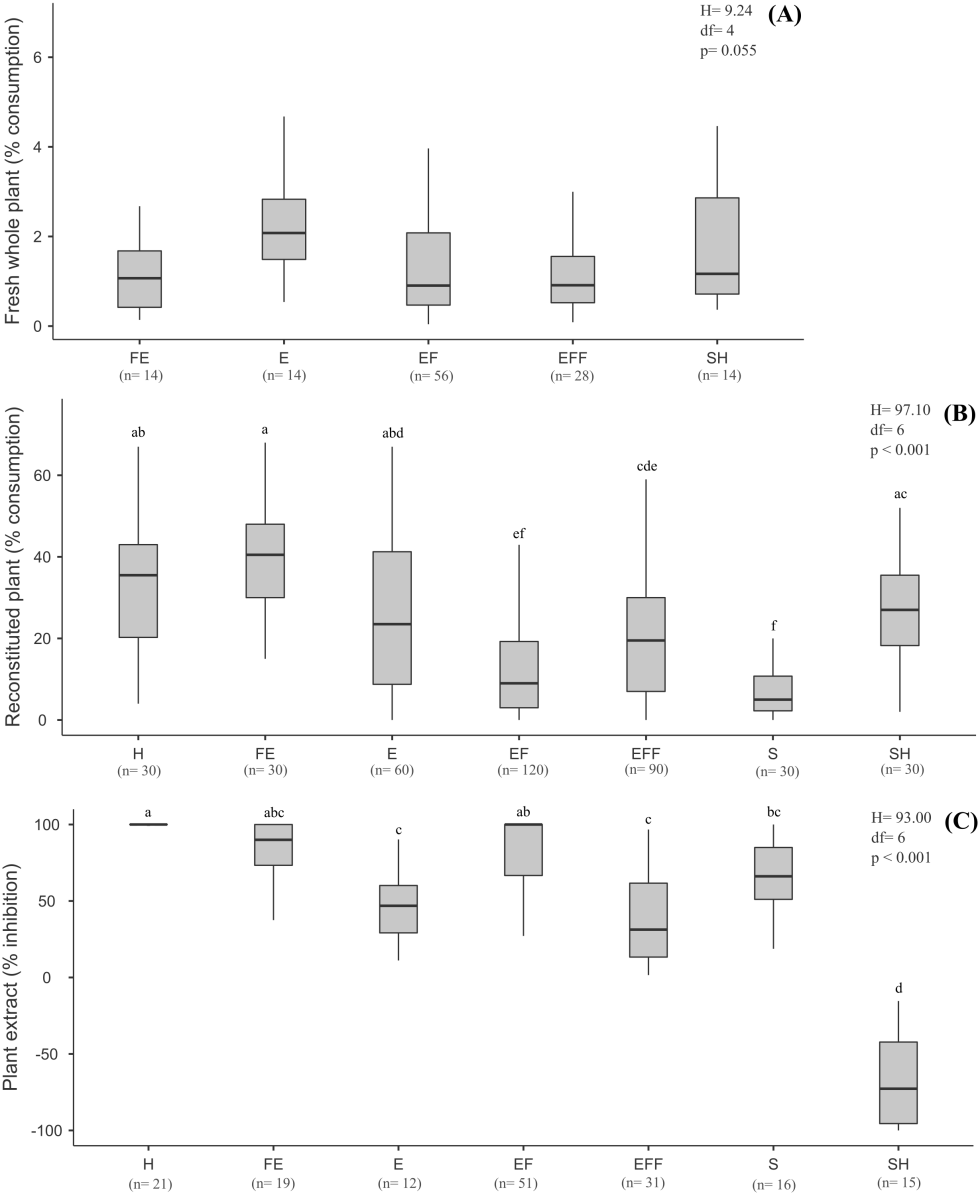
Results of the choice feeding assays results with of *B. glabrata*, analyzed according to the plant life-forms: (A) Susceptibility of nine fresh whole plants; (B) consumption of thirteen reconstituted aquatic plants; and (C) defensive effect of ten plant extracts. The same letters on bars indicate the treatments are not significantly different. Lower and upper box boundaries 25th and 75th percentiles, respectively, line inside box median and lower and upper error lines 10thand 90t percentiles, respectively. H = Helophyte, FE = Fixed emergent, E = Emergent, EF = Emergent floating, EFF = Emergent Free Floating, S = Submerged, SH = Submerged to Helophyte.

There was no significant pattern in terms of plant biomass consumed according to plant life-forms (*H* = 349.94, *df* = 4, *p* = 0.055) (Fig. 2A), though *B. glabrata* had consumed more emergent plants (2.08%) than emergent floating plants (0.90%).

### Consumption of reconstituted aquatic plants by *B. glabrata*

When we eliminated interspecific differences arising from physical/structural characteristics, but retained the taste, nutritional and chemical properties of the thirteen aquatic plants by offering reconstituted palnts as artificial food to *B. glabrata*, we obtained different results from those observed for fresh plants (Fig. 1A). Again, the gastropod consumed all 13 plants (Fig. 1B), but in significantly different amounts (*χ*^2^ = 144.36, *df* = 12, *p* < 0.001, Tukey test). Moreover, distinct groupings were not observed and the hierarchy of diminishing preference was quite different: *T. domingensis* > *X. brevifolia* > *N. pulchella* >  *E. interstincta* > *O. salzmannii* > *H. nymphoides* > *C. thalictroides* > *P. stratiotes* > *N. humboldtiana* > *S. auriculata* > *E. azurea* > *U. foliosa* > *P. illinoensis*. For instance, *Eichornia azurea* that was highly consumed as a fresh plant was one of the least preferred as a reconstituted food. However, *P. illinoensis* remained unpalatable the least palatable (3.00% consumption, median value), suggesting it harbor a component that exerts an inhibitory effect on consumption by *B. glabrata*. In contrast, *T. domingensis* that was one of the least susceptible to consumption as a fresh plant, was the most preferred (% consumption, median value) as reconstituted food, indicating that it possesses structural components that deter consumption by this gastropod.

Considering the life-forms of the plants reconstituted as artificial food (Fig. 2B), we noted a significant decreasing preference by *B. glabrata* (*H* = 197.10, *df* = 6, *p* < 0.001, Tukey test): fixed emergent > helophyte > submerged to helophyte > emergent > emergent free floating > emergent floating > submerged. Accordingly, *B. glabrata* appears to exhibit a preference for consuming aquatic plants that occupy the marginal areas of the lagoons from which they were collected.

### Defensive capabilities of the plant extracts

The nine extracts of aquatic plants we assayed exhibited significantly different defensive effects (*H* = 101.10, *df* = 8, *p* < 0.001, Tukey test) against consumption by *B. glabrata* (Fig. 1C), with inhibition (%) ranging from 100% (*P. illinoensis*) to 13.3% (*S. auriculata*), and with a hierarchy of diminished inhibition of: *P. illinoensis* > *X. brevifolia* > *T. domingensis* > *E. azurea* > *N. humboldtiana* > *U. foliosa* > *E. interstincta* > *P. stratiotes* > *S. auriculata.* In contrast, the extract from *O. salzmannii* significantly stimulated consumption by the gastropod. When we focused in plant life-forms, we found significant differences between species in terms of extract-mediated herbivory inhibition (*H* = 93.00, *df* = 6, *p* < 0.001, Tukey test), with the following hierarchy (Fig. 2C): emergent floating = helophyte > fixed emergent > submerged > emergent > emergent free floating. Chemicals from submerged and helophyte plants stimulated the consumption. For the emergent floating plant *P. illinoensis,* this result reiterates that obtained in previous assays about susceptibility to consumption by *B. glabrata* (Figs. 1A and 2A), as well as their consumption as reconstituted food (Figs. 1B and 2B), since in both they were the least consumed food items.

### Physical, nutritional and chemical properties of the aquatic plants

The physical, nutritional and general chemical defense properties we assessed varied significantly among the plant species and also when analysed to life-forms (Table 2) Dry mass, ash, lipid, total nitrogen, protein, and phenol levels varied from five-fold (g% nitrogen) to almost thirty-fold (% lipid). *Salvinia auriculata* displayed the lowest percentage dry mass, whereas *X. brevifolia* scored highest in this respect, reflecting their life-form as submerged and helophyte plants, respectivley. Ash (%) was lowest in *E. interstincta* (7.10 ± 0.18%) and highest in *P. stratiotes* (39.42 ± 0.47%), and more generally was lower among emergent free-floating plants. Lipid contents dysplaied the widest variation, ranging from 1.16 ± 0.07% in *E. interstincta* to 20.52 ± 1.03% in submerged to helophyte *O. salzmannii.* This later species also presented the lowest levels of nitrogen and protein (0.46 ± 0.02 g% N and 2.85 ± 0.13 g% P, respectively) in marked contrast to *N. pulchella* that harbored the highest amounts (2.73 ± 0.03 g% N and 17.08 ± 0.16 g% P). Phenol levels also showed considerable amount variation, ranging from 3.84 ± 0.02 mg GAE/g extract in *X. brevifolia* to 77.98 ± 0.09 mg GAE/g extract in *T. domingensis*, representing helophyte and fixed emergent plants, respectively.

**Table 2:** Physical (% dry mass ash), nutritional (g% nitrogen, g% protein and % lipid), and general chemical defense (phenol content - mg GAE/g extract) properties of the studied aquatic plants. Values are means ± standard deviations. Different letters indicate statistically significant differences.

	**Dry mass**	**Ash**	**Lipid**	**Nitrogen content**	**Protein content**	**Phenol** **content**
***Plant species***						
*X. brevifolia*	39.42 ± 0.47^a^	16.76 ± 0.27^a^	–	0.94 ± 0.03^a^	5.85 ± 0.19^a^	3.84 ± 0.02^a^
*E. interstincta*	28.24 ± 0.43^b^	7.10 ± 0.18^b^	1.16 ± 0.07^a^	1.03 ± 0.04^a^	6.42 ± 0.25^a^	49.07 ± 0.06^b^
*T. domingensis*	16.18 ± 0.21^c^	8.33 ± 0.20^c^	6.65 ± 0.81^bc^	0.54 ± 0.04^b^	3.29 ± 0.22^b^	77.98 ± 0.09^c^
*P. illinoensis*	7.50 ± 0.13^d^	14.88 ± 0.09^d^	6.62 ± 0.20^bc^	2.00 ± 0.16^c^	12.48 ± 0.97^c^	63.99 ± 0.12^d^
*H. nymphoides*	6.63 ± 0.05^e^	25.36 ± 0.21^e^	7.85 ± 0.76^bd^	1.73 ± 0.10^d^	10.79 ± 0.63^d^	–
*N. humboldtiana*	9.42 ± 0.58^f^	9.36 ± 0.07^f^	1.44 ± 0.06^a^	1.62 ± 0.08^de^	10.15 ± 0.50^de^	70.85 ± 0.10^e^
*N. pulchella*	8.72 ± 0.18^g^	10.38 ± 0.17^g^	1.84 ± 0.05^a^	2.73 ± 0.03^f^	17.08 ± 0.16^f^	–
*E. azurea*	9.32 ± 0.33^h^	22.97 ± 0.33^h^	5.50 ± 0.50^c^	0.97 ± 0.02^a^	6.06 ± 0.13^a^	28.98 ± 0.02^f^
*S. auriculata*	3.05 ± 0.09^i^	25.36 ± 0.17^e^	9.48 ± 0.61^d^	1.62 ± 0.07d^e^	10.13 ± 0.44^d^	41.77 ± 0.04^g^
*P. stratiotes*	–	39.35 ± 0.46^i^	6.40 ± 0.41^bc^	2.46 ± 0.03^g^	15.35 ± 0.16^g^	60.11 ± 0.04^h^
*C. thalictroides*	–	15.87 ± 0.15^j^	31.65 ± 1.52^e^	1.55 ± 0.03^eh^	9.67 ± 0.16^e^	–
*O. salzmannii*	–	15.35 ± 0.09^j^	20.52 ± 1.03^f^	0.46 ± 0.02^b^	2.85 ± 0.13^b^	55.72 ± 0.05^i^
*U. foliosa*	3.49 ± 0.07^j^	21.39 ± 0.54^l^	11.70 ± 0.70^g^	1.68 ± 0.11^dh^	10.52 ± 0.66^dh^	47.04 ± 0.07^j^
***Habit (life-form)***						
SH	–	15.35 ± 0.09^b^	20.52 ± 1.03^a^	0.46 ± 0.02^ab^	2.85 ± 0.13^ab^	55.72 ± 0.05^a^
H	39.42 ± 0.46^a^	16.76 ± 0.27^b^	–	0.94 ± 0.03^ab^	5.85 ± 0.19^ab^	3.84 ± 0.01^b^
FE	16. 18 ± 0.21^b^	8.33 ± 0.19^b^	6.56 ± 0.81^ab^	0.53 ± 0.03^ab^	3.29 ± 0.22a^b^	77.98 ± 0.09^a^
E	28.24 ± 0.42^c^	11.48 ± 4.80^b^	16. 40 ± 17.73^a^	1.28 ± 0.29^a^	8.04 ± 1.79^a^	49.07 ± 0.06^a^
EF	8.74 ± 0.85^d^	14.40 ± 5.60^b^	3.85 ± 2.36^b^	1.83 ± 0.67^a^	11.44 ± 4.18^a^	54.61 ± 19.45^a^
EFF	4.84 ± 1.97^e^	30.02 ± 7.00^a^	7.91 ± 1.43^ab^	1.93 ± 0.40^a^	2.49 ± 0.83^a^	50.94 ± 10.04^a^
S	3.49 ± 0.07^j^	21.39 ± 0.54^ab^	11.70 ± 0.70^ab^	1.68 ± 0.10^a^	10.52 ± 0.38^a^	47.04 ± 0.07^a^

### Relationships between plant traits and its consumption by *B. glabrata*

**Figure 3:**
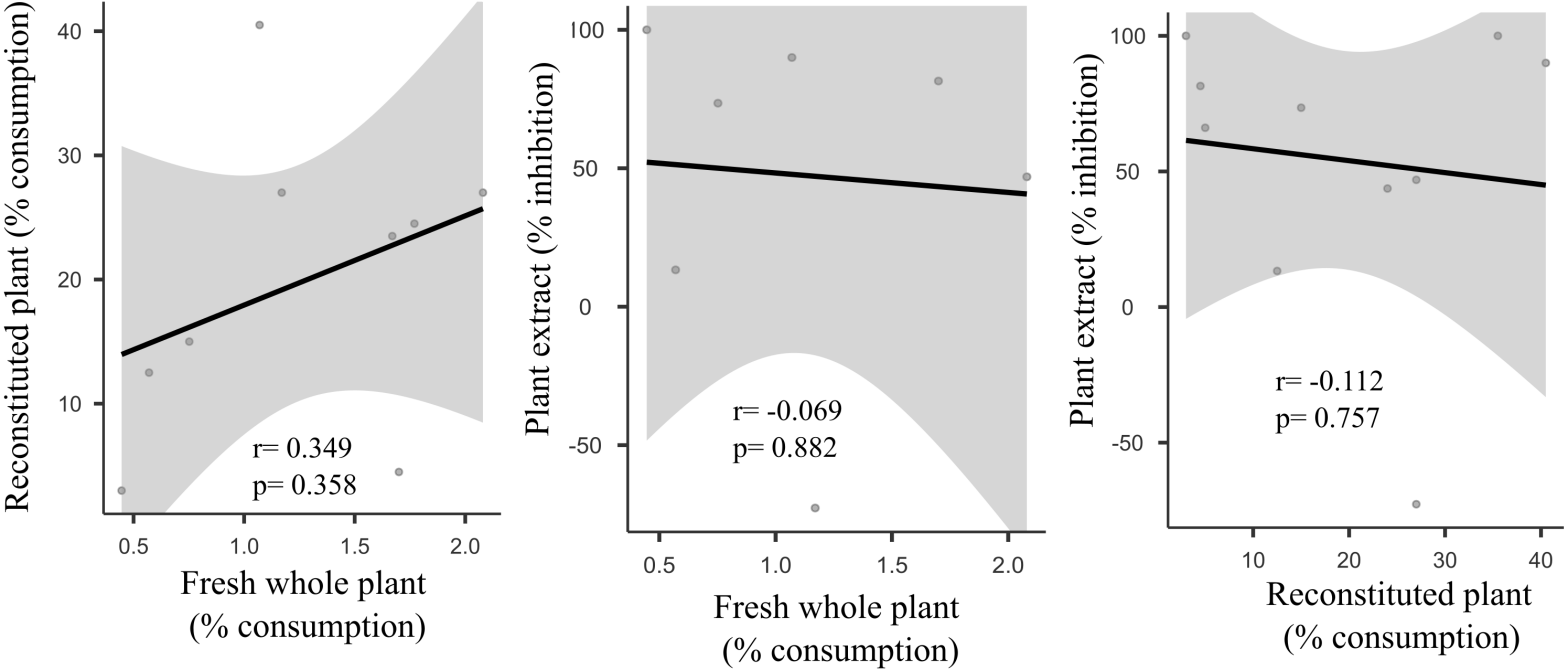
Pearson correlation matrix pair between relationships between percentage consumption of whole plants, artificial foods and extract-mediated inhibition of consumption. r = Pearson correlation coefficient. In bold is highlighted the significant correlations.

**Figure 4:**
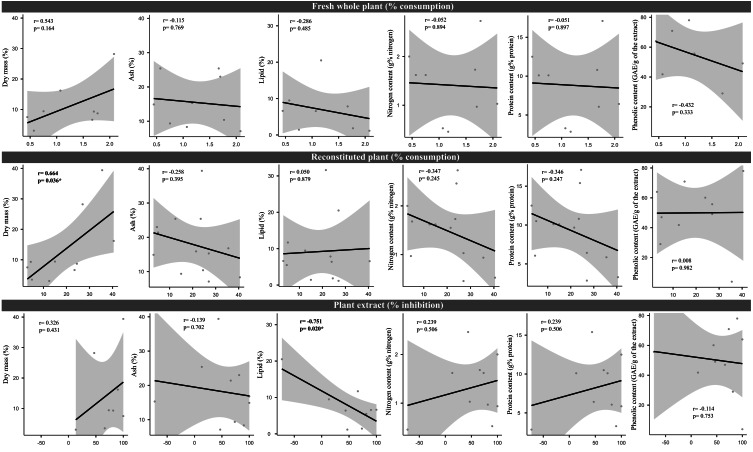
Pearson correlation matrix pair about relationships between percentage consumption of whole plants, artificial foods, extract-mediated inhibition of consumption and the various physical, nutritional and chemical traits of the plant species. r = Pearson correlation coefficient. In bold is highlighted the significant correlations.

**Figure 5:**
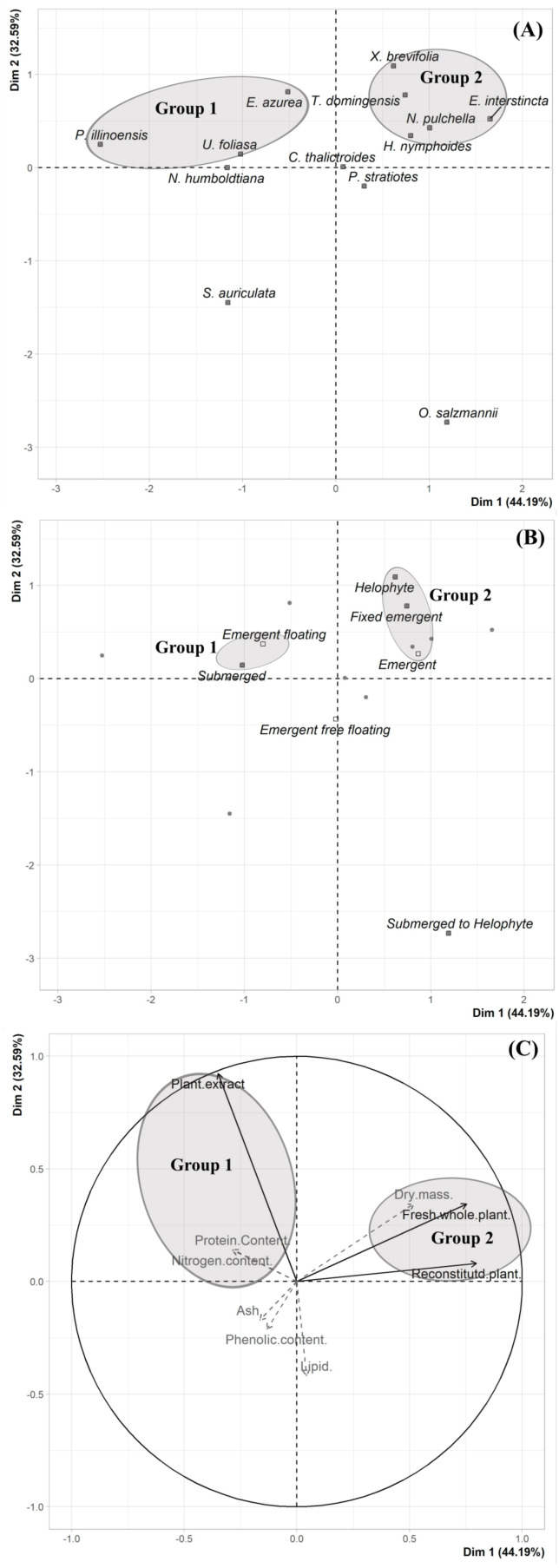
Results of Principal Component Analysis (PCA) separated by Dim 1 = PC1 and Dim 2 = PC2. (A) Two-dimensional score plot of the aquatic plant species. (B) Two-dimensional score plot of the different plant life-foms. (C) Two-dimensional loading plot. Black arrows represent the mains quantitative variables (choice feeding assays) and gray arrows represent the supplementary quantitative variables (physical, nutritional and chemical traits).

We did not uncover significant correlations between the susceptibility to consumption of fresh plants consumption of reconstituted plants, and the defensive capacity of the corresponding plant extracts (Pearson correlation coefficients, Fig. 3). Likewise, no significant relationships were observed between these three parameters and most of the various analyzed plant traits (ash, lipids, protein, total nitrogen and phenol) (Fig. 4), apart from a negative correlation between % extract inhibition and % lipids (R_Pearson_ = −0,75; *p* = 0.02). When we observe the PCA result, the first two axes of our PCA analysis summarized 76.78 % of the data variation (PC1 = 44.19 % and PC2 = 32.59 %, Fig. 5A, Fig. 5B and Fig. 5C). The ordination of the samples (Fig. 5A and Fig. 5B) and variables (Fig. 5C) formed by these axes support our results described in previous sections. Overall, the PCA divided the samples into two groups: group 1 in the PC1 negative and PC2 positive quadrant; and group 2 in the PC1 positive and PC2 positive quadrant. In terms of species, the ordination of the of species sample (Fig. 5A), P. illinoensis, E. azurea and U. foliosa and formed group 1, and E. interstincta, X. brevifolia, T. domingensis, N. pulchella and H. nymphoides formed group 2. Ordination according to plant life-form (Fig. 5B) discriminated two groups, i.e., emergent floating and submerged plants (group 1) and helophyte, fixed emergent and emergent plants (group 2). The ordination of the variables (loading, Fig. 5C) revealed that the group 1 encompassing plants whose extracts exhibited higher defensive activity against B. glabrata, exemplified by P. illionensis. The group 2 (Fig. 5C) represents the plants most susceptible to or preferred by B. glabrata as fresh or reconstituted food, in addition to revealing a strong correlation between dry mass and both consumption of fresh and reconstituted plants.

PCA analysis showed that the first two axes summarized 76.78% of the data variation (PC1 = 44.19% and PC2 = 32.59%, Figs. 5A, 5B, 5C). The ordination of the samples (Figs. 5A and 5B) and the variables in the space (Fig. 5C) formed by these axes reiterated the previous analysis of the data of the percentage of consumption of whole plants, reconstituted plants, inhibition of the extracts, and the several analyzed plant physical, nutritional and chemical traits and their correlations. Overall, the PCA divided the samples into two main groups: group 1 in the PC1 negative and PC2 in positive quadrant; and group 2 in the PC1 positive and PC2 positive quadrant. As main results, in the ordination of the species sample (Fig. 5A), it is possible to observe a group formed by *P. illinoensis, E. azurea, U. foliosa* and *N. humboldtiana* (group 1); and another group formed by *E. interstincta, X. brevifolia, T. domingensis, N. pulchella* and *H. nymphoides* (group 2). The ordination by life-form (Fig. 5B) discriminated two large plant groups: emergent floating and submerged plants (group 1) and helophyte, fixed emergent and emergent plants (group 2). The ordination of the variables (Fig. 5C) revealed that the group I includes those plants whose extracts exhibited higher defensive action against *B. glabrata*, mainly *P. illinoensis*. In contrast, group 2 (Fig. 5C) represents those plants most susceptible to or preferred by *B. glabrata* when offered to this gastropod either as a whole or as a reconstituted plant.

### Chemical profile of *P. illinoensis* extract

The extract from *P. illinoensis* proved the most defensively capable against *B. glabrata* among the tested plants (Fig. 1C), so we further investigated its chemical profile by ^1^H NMR. Characteristic signals of fatty acids were observed in the aliphatic region, *i.e.,* between *δ* 0.86 parts per million (ppm) and *δ* 1.70 ppm. An *α*-CH_2_ acyl group was observed at *δ* 2.32 (m), and strong overlapping resonance signals of methylene groups were also observed at *δ* 1.25 (sl). Additionally, we detected signals of exocyclic methylene (*δ* 4.0–5.0 ppm), methyl groups (*δ* 0.5–1.5 ppm) and hydrogen bonded to the sp^2^ carbon of furane or lactone moieties (*δ* 6.09–7.11 ppm), which are characteristics of labdane diterpenes commonly reported in species of the *Potamogeton* genus (Waridel et al., 2003) were also detected.

## Discussion

In this study, we investigated the susceptibility of various aquatic plants (as fresh and reconstituted artificial foods), to consumption by the generalist gastropod *B. glabrata,* and also explored the contribution of extracts as a chemical defense and several other plant traits to this plant-herbivore interaction.

We revealed rather indiscriminate consumption of all smapled plant species despite their varied life-forms, including emergent free floating (*H. nymphoides*, *S. auriculata*, *P. stratiotes*), emergent floating (*E. azurea*, *N. pulchella*, *P. illinoensis*), emergent (*E. interstincta* and *C. thalictroides*), fixed emergent (*T. domingensis*), submerged to helophyte (*O. salzmannii*), and helophyte (*X. brevifolia*). This outcome contrats with several previous studies that have reported how the life-form of aquatic plants impacts their relationships with consumers (Lodge, 1991; Smolders et al., 2000; Bakker et al., 2016). Submerged, emergent, and floating-leaved plants are subjected to high herbivory pressure by diverse consumers (Lodge, 1991), implying that different aquatic plant life-forms predispose to different consumer interactions. For example, both emergent and shallow submerged aquatic plants would be accessible to terrestrial herbivores, whereas obligate aquatic herbivores could only consume submerged plants (Gross & Baker, 2012). Previously, herbivory rates were found to be lower on emergent aquatic plants relative to submerged ones (Bakker et al., 2016), and emergent plants appear to be less susceptible to consumption by invertebrates than submerged and floating forms (Lodge, 1991). An analysis of phenolic contents in 40 aquatic and semi-aquatic plants revealed lower levels in submerged species compared to emergent or floating ones (Smolders et al., 2000), although that pattern was not observed for the 10 species we analyzed here.

The dry mass contents of aquatic plants have been negatively correlated with consumption rates (*e.g.*, Elger & Willby, 2003; Wong et al., 2010) and structural defenses are more commonly found among upland plants than wetland ones (Lodge, 1991), though either of these features explain preferential consumption of aquatic plants (Prejs, 1984). Among the plants with higher amounts of dry matter considered in our study, *E. interstincta*, *X. brevifolia* and *T. domingensis*, only in the emergent *T. domingensis* might this trait play any importance in its consumption, albeit perhaps not statistically significantly. *Typha domingensis* was largely rejected as food by *B. glabrata* when presented as fresh plant, but it was the most consumed when presented as reconstituted artificial food. Interestingly, the three emergent species most consumed by *B. glabrata*, *E. interstincta* > *N. pulchella* > *H. nymphoides*, exhibited low levels of ash and high amounts of aerenchyma (T. Konno, 2021, pers. comm.), with this latter being an important characteristic facilitating the plant fluctuation, but potentially rendering them more susceptible to herbivory.

In general, submerged plants need fewer structural components, but generate higher nitrogen levels and suffer higher rates of herbivory relative to emergent species (Bakker et al., 2016). In our experiments using reconstituted plants, the submerged species *O. salzmannii* that displays low nutritional value (low amounts of nitrogen and protein) was moderately consumed by *B. glabrata*, but its extract stimulated the consumption by the gastropod. In contrast, *U. foliosa* has high levels of nitrogen but was largely not consumed by *B. glabrata*.

We found no evidence of a relationship between the susceptibility to consumption of fresh plants and their reconstituted artificial foods or for the defensive action of their extracts on this gastropod. These results support consumption of the assayed plants by *B. glabrata* being rather generalized, as they been reported for a variety of other aquatic consumers, such as gastropods (Sheldon, 1987), crayfishes (Seroll & Coler, 1975; Cronin et al., 2002), grass carp (Wiley, Pescitelli & Wike, 1986; Bowers, Pauley & Thomas, 1987; Parker & Hay, 2005), and mosse (Fraser, Chavez & Paloheimo, 1984).

The evidence clearly points to *P. illinoensis* being the least susceptible to consumption, either as fresh plant or as reconstituted food, and its extract showed the highest percentage of inhibition among all the species we studied. This outcome supports several previous studies showing that showed that aquatic plants are commonly unpalatable and contain a variety of secondary metabolites (*e.g.*, terpenes, phenols and alkaloids) known for their ecological role as chemical deterrents against herbivory (Ostrofsky & Zettler, 1986; Kubanek et al., 2001; Cronin et al., 2002; Prusak, O’Neal & Kubanek, 2005). *Potamogeton illinoensis* is a known producer of secondary metabolites (Zhang et al., 2011) and its close relative *Potamogeton pectinatus* exhibit algicidal and allelopathic activity (Waridel et al., 2003). Consequently, these chemicals likely exhibit ecological roles as deterrent to the activity of herbivores such as the gastropod *B. glabrata*.

Several other species we studied also presented high percentage of inhibition of *B. glabrata* consumption, such as *X. brevifolia*, *T. domingensis*, *E aurea*, *N. humboldtiana*, *U. foliasa*, *E. interstincta* and *P. stratoides*. Several of these or related species are known to produce secondary metabolites. For example, *T. domingensis* is known to produce phenols and fatty acids (Gallardo-Williams et al., 2002), and an extract from it inhibits feeding by crayfish (Bolser et al., 1998). Species of *Xyris* also produce defensive chemicals, such as the isocoumarins of *X. indica* (Ruangrungsi et al., 1995), the anthraquinones of *X. semifuscata* (Fournier et al., 1975) and the flavonoids from *X. itatiayensis*, *X. longiscapa* and *X. obtusiuscula* (Varanda, Rondinoni & dos Santos, 2002). Phenols, many displaying allelopathic activity, have been identified in species of *Eleocharis* (Frank & Dechoretz, 1980; Zaman et al., 2018), and *Eichornia crassipes* produces phenalenone compounds and sterols (Lalitha, Sripathi & Jayanthi, 2012). Anthraquinones (Siva et al., 2012) and cyclic peptides (Seydel & Dörnenburg, 2006) have been identified from the genus *Oldenlandia*. Similarly, several classes of secondary metabolites have been detected in *Ceratopteris thalictroides* (Smitha & Vadivel, 2019), and autoinhibitory compounds were also identified in *Salvinia molesta* (Li et al., 2016). Chemicals from *Potamogeton maackianus* were shown to exert allelopathic activity against the microalga *Selenastrum capricornutum* (Zhang et al., 2011), and *ent*-labdane diterpens from *P. pectinatus* had the same effect on *Raphidocelis subcapitata* (Waridel et al., 2003). Interesting, we identified en-labdane diterpenes in the *P. illinoensis* extract we assessed by ^1^H NMR. Alkaloids have been detected in *P. amplifolius* (Ostrofsky & Zettler, 1986), and high levels of phenols were found in the propagules of three species of *Potamogeton* (*P. gramineus*, *P. nodosus* and *P. pectinatus*) (Spencer & Ksander, 1994), proving evidence that species of this genus protect themselves from herbivory through chemical defense. Based on our findings, we suggest that, apart from *H. nymphoides*, all other assayed species are producers of chemicals that could act as chemical deterrents, resulting in those species being less susceptible to consumption by *B. glabrata*.

The defensive function against herbivory of phenols in plants is well knwon (Lodge, 1991). Almost all of the species we assayed produce phenols, such as *E. interstincta*, *N. humboldtiana*, *O. salzmannii*, *P. stratiotes* and *T. domingensis*, but these plants were well-consumed by *B. glabrata*. The only exception was *P. illinoensis*, representing the plant least consumed by *B. glabrata*, but we found it harbored other defensive substances, such as diterpenoids.

Plant nutritional value and chemical defense capability are the primary traits determining plant palatability (Cronin et al., 2002; Sotka et al., 2009). Aquatic plants of high nutritional value should be favored by generalist herbivores such as crayfish and apple snails (Ampullariidae) because eating them would maximize fitness, yet there is a trade-off between feeding on nutrient-rich food and ingesting defensive plant metabolites (Lodge, 1991). However, two plant species with the highest nutritional values (nitrogen and protein) that we studied, *N. pulchella* and *P. stratiotes*, were not the most preferred by *B. gabrata* in our feeding assays. Floating plants, typically exhibit higher nitrogen contents than other aquatic plants (Lodge, 1991), but in for four of the floating plants we analysed (*H. nymphoides*, *N. humboldtana*, *N. pulchella* and *P. stratiotes*), intermediate-to-high nitrogen and protein levels were detected, but they were not preferred food items of *B. glabrata.*

Despite all the plant parameters assayed in this study, we did not detect prevalence of one protective or attractive factors that could explain the plant-herbivore relationship with *B. glabrata*, despite all of the traits we analyzed being known to influence feeding by invertebrates on aquatic plants. This outcome may not be surprising, since the consumption of aquatic plants may be unrelated to their physical and chemical characteristics (Lodge, 1991). However, we believe that further studies on the relationships of aquatic plants with herbivores are still needed, necessitating an integrative approach as applied herein and assessing other types of herbivores. Although gastropods exert an important top-down control on aquatic plants through grazing (Lodge, 1991; Newman, 1991; Lodge et al., 1998), this effect is dispersed across the food web due to their generalist diet. Despite identifying a hierarchy of plant consumption by *B. glabrata*, its lack of a clear preference implies that this gastropod makes its food choice based on multiple plant traits, thereby exerting a diffuse effect on these plants in their natural environment.

## Supplemental Information

10.7717/peerj.12031/supp-1 Supplemental Information 1 Raw data

## Additional Information and Declarations

### Field Study Permissions

The following information was supplied relating to field study approvals (i.e., approving body and any reference numbers): Instituto Chico Mendes de Conservação da Biodiversidade approved this study (SISBio 30299).

### Data Availability

The following information was supplied regarding data availability: The raw data are available in the Supplemental File.
